# Semantic Sentiment Classification for COVID-19 Tweets Using Universal Sentence Encoder

**DOI:** 10.1155/2022/6354543

**Published:** 2022-10-05

**Authors:** Ibrahim Eldesouky Fattoh, Fahad Kamal Alsheref, Waleed M. Ead, Ahmed Mohamed Youssef

**Affiliations:** ^1^Computer Science Department, Faculty of Computers and Artificial Intelligence, Beni-Suef University, Beni-Suef, Egypt; ^2^Information Systems Department, Faculty of Computers and Artificial Intelligence, Beni-Suef University, Beni-Suef, Egypt; ^3^Faculty of Education, Helwan University, Helwan, Egypt

## Abstract

The spread of data on the web has increased in the last twenty years. One of the reasons is the appearance of social media. The data on social sites describe many real-life events in our daily lives. In the period of the COVID-19 pandemic, a lot of people and media organizations were writing and documenting their health status and the latest news about the coronavirus on social media. Using these tweets (sentiments) about the coronavirus and analyzing them in a computational model can help decision makers in measuring public opinion and yielding remarkable findings. In this research article, we introduce a deep learning sentiment analysis model based on Universal Sentence Encoder. The dataset used in this research was collected from Twitter, and it was classified as positive, neutral, and negative. The sentence embedding model determines the meaning of word sequences instead of individual words. The model divides the dataset into training and testing and depends on the sentence similarity in detecting sentiment class. The obtained accuracy results reached 78.062%, and this result outperforms many traditional ML classifiers based on TF-IDF applied on the same dataset and another model based on the CNN classifier.

## 1. Introduction

As a result of its significant impact on people's day-to-day lives around the globe, the emergence of COVID-19 sparked a global wave of anxiety and fear. Governments have long employed lockdowns and social isolation against mental health of the populace. Keeping a track of mental health of individuals throughout a variety of events and topics will be essential for making appropriate decisions.

The globe was in terrible condition as a result of the sudden spread of the coronavirus. The COVID-19 epidemic was claiming the lives of people across the planet. This sickness has had profound effects on people in both explicit and tacit ways.

Twitter and other social media platforms are considered huge data repositories that may be used to collect data for studying human psychology and behavior so as to get a deeper understanding of psychology and health. Various social media channels have a significant impact on boosting public knowledge about the disease's importance and advocating preventive measures among community members [1]. Sentiment analysis is the “process of analyzing text with the help of machine learning and natural language processing (NLP) methods to identify the polarity of text” for a better emotional understanding of individuals or social groups. Social media platforms provide several challenges. Social media, as a rapidly increasing online forum for exchanging opinions and ideas, offers many chances for decision makers to better comprehend public sentiment [2, 3]. Users can quickly convey their sentiments on social media [4]. Many academics from all over the world have looked into the use of social media in a variety of sectors, with one of the most important being their involvement during disease outbreaks [5]. Because created data are very dynamic and relevant for real-time trends, social media analysis is a promising field [5]. This method can provide a low-cost, quick, and effective public health monitoring system on a large scale [2, 6]. Facebook, Twitter, Reddit, Instagram, and news forums are some of the most well-known social media sites that are heavily used in sentiment analysis [7]. Even these priceless assets face several obstacles that could stymie their final implementation.

Researchers in the field of sentiment analysis highlighted concerns and obstacles of the social media platform in terms of trustworthiness and authenticity [5, 8]. Other researchers talked about difficulties of social media platforms due to the cadence of content capability limitations [9], possible exaggeration [6], and challenges in comprehending emotions, particularly in situations such as epidemics [4] or different sources [7, 9].

Issues relating to the community individuals or parties from other fields or agencies are welcome to join the community in this section. Two parties were primarily targeted when scanning the issues related to sentiment analysis and infectious diseases and outbreaks, in addition to viruses, epidemics, and pandemics: (1) decision makers and (2) the scientific community. In particularly dangerous circumstances, such as disease outbreaks, decision makers face obstacles such as detecting people's attitudes on a subject and their views towards public health policy decision-making [4, 10, 11]. Furthermore, this problem will force decision makers to act quickly to prevent the spread of an event or even quarantine confirmed instances of infectious diseases [6, 12, 13]. In addition to decision makers, the scientific research community plays a crucial role in continuing to fight outbreaks through sentiment analysis; but even so, fully utilizing the scientific community's abilities remained unclear due to the shortage of scientific reference works [6], the availability of studies in specific languages only (Ji et al.), or the lack of relevant studies with the most emerging advancements [12]. The government has instituted public plans (such as “stay home” and “social separation”) and implemented travel restrictions in response to the pandemic. If social media had properly guided us through this perilous situation, it would have been favorable.

Contrary to popular belief, it has been discovered that people were using social media to spread fake drugs or incorrect information [14]. Because of the lockdown, millions of people used social media for the first time to stay informed. It would be preferable if accurate information could be disseminated and people could be kept informed about this dreadful sickness that had engulfed the entire world. It has produced an uncomfortable situation among people by disseminating inappropriate content about COVID-19, leading to mental disturbances. Many people believe that utilizing social media is detrimental to one's health [15]. Coronavirus is airborne and can linger on surfaces for hours, according to facts. It is easy to target older adults, it causes breathlessness, it causes mortality in a matter of days, it is incurable, and it is making rounds on social media at an unusually fast rate [16, 17].

In this research study, we provided a smart solution that can help in the case of the pandemic to analyze tweets (sentiments) written by people about what they feel with the symptoms of the disease, and using a computational model can assist decision makers in measuring public opinion and yielding remarkable findings. In this research study, a deep learning sentiment analysis model based on Universal Sentence Encoder is applied to a dataset collected from Twitter about coronavirus. The main contribution of this research is that the model is based on the semantic similarity of sentences not on their syntactic structure or word embeddings that constitute the tweet.

## 2. Related Work

Along with analyzing coronavirus-related searches using Google Trends, Basiri et al. [1] proposed a new technique based on the merging of four deep learning and one traditional supervised machine learning model for sentiment classification of tweets from eight countries. Chandra and Krishna [18] introduced Twitter-based sentiment analysis for COVID-19 in India using the Twitter dataset to develop LSTM, BD-LSTM, and BERT models. Findings indicate that a vast majority of tweets have been positive, with high levels of hopefulness, during the increase of novel COVID-19 cases, and that the number of tweets has significantly decreased as the epidemic has reached its peak. Rustam et al. [19] evaluated the effectiveness of different machine learning classifiers utilizing their proposed feature set that comprised bag-of-words and term frequency-inverse document frequency. Using the long short-term memory (LSTM) architecture of the deep learning model, they classified tweets as positive, neutral, and negative. The results demonstrated that extratree classifiers outperformed all other models with an accuracy score of 0.93. The LSTM was less accurate than other machine learning classifiers. The study conducted by Chakraborty et al. [20] demonstrated word cloud or word frequency in tweets. They proposed a model using deep learning classifiers with an accuracy of up to 81%. As an additional method, they proposed using a fuzzy rule base based on the Gaussian membership function to accurately identify sentiments from tweets. This model achieved a maximum allowable accuracy of 79%. Chakraborty et al. [21] developed an experimental approach to predict sentiment on COVID-19 data so as to analyze the reactions of people on Twitter. They used an evolutionary classification-based LSTM model followed by the n-gram analysis; the model achieved an overall accuracy of 84.46%. Kaur et al. [22] proposed a hybrid heterogeneous support vector machine (H-SVM) algorithm that performed the sentiment classification of COVID tweets to determine the impact of Twitter data analysis on the mental health status of people. Algorithms classified them as positive, negative, and neutral sentiment scores. They compared their algorithm with RNN and SVM. Manguri et al. [23] applied sentiment analysis on Twitter data related to worldwide COVID-19 outbreaks using the python Text blob library which uses the naïve Bayes model to discover sentiment emotion for coronavirus. Nemes and Kiss [24] used a recurrent neural network (RNN) for Twitter sentiment classification based on COVID-19 to classify emotions that occurred within the pandemic, marking them with an articulated class of emotional strength (weakly positive/negative, strongly positive/negative) emotions.

Zhu et al. [25] evaluated the public sentiments of microblog text subjects using latent Dirichlet allocation (LDA), one of the most effective technologies in social media semantic mining. Yin et al. [26] proposed an approach to analyze the topic and sentiment dynamics of COVID-19-related posts on social media. The goal of the model was to dynamically recognize trending topics and sentiment polarities associated with them. They used the dynamic topic model (DTM) and VADER sentiment lexicon tools. Dubey [27] collected tweets related to COVID-19 from twelve countries to analyze how the citizens of different countries deal with the situation. They used the NRC emotion lexicon and word cloud to compare countries according to scores of emotions (anger, anticipation, disgust, fear, joy, sadness, surprise, and trust) and sentiments (positive and negative). Shahi et al. [28] proposed a sentiment classifier for peoples' tweets on COVID-19, which is available in the Nepali language. They provided a hybrid feature extraction based on both TF-IDF and fast text embedding text representation methods and applied nine ML classifiers. The results showed that the hybrid feature extraction method outperformed each feature extraction. Sitaula et al. [29] also introduced a model for analyzing people's sentiments on COVID-19 tweets by introducing a feature extraction method based on three text representations. They proposed three different CNN model classifiers, and at last, they ensembled the three CNNs that reached 68.7% accuracy.

As can be seen that the abovementioned works adopted different machine learning and deep learning algorithms based on word embedding, here we propose the semantic similarity model that applies sentence-based embedding using Universal Sentence Encoder for embedding and training tweets and then classifying the new ones.

## 3. Universal Sentence Encoder

**Figure 1:**
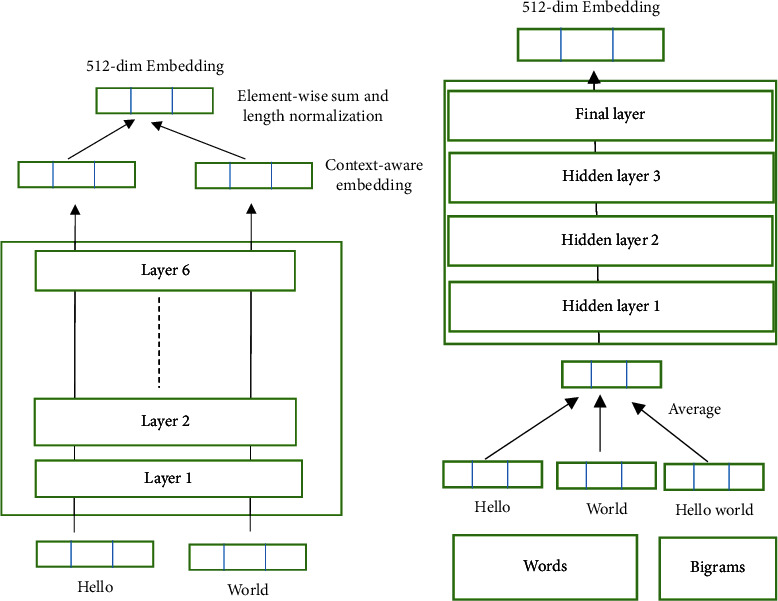
Two sentence encoder models: (a) transformer encoder and (b) DAN Encoder.

Textual input is encoded into high-dimensional vectors by Universal Sentence Encoder models, which can be used for a variety of NLP applications. It was first mentioned in the article [30]. Modeling the meaning of word sequences rather than individual phrases is required by the encoders employed in such models. In addition to single words, the models are trained and optimized for content that is longer than just a single word, such as sentences, phrases, or paragraphs. The model encoders are divided into two types: one that employs a transformer architecture and the other that uses a deep averaging network (DAN) as shown in Figure 1. These models provide a static 512-dimensional vector dimensional embedding representing the input strings when the input is the variable-length English text. To generate sentence embedding using the transformer model, the coding subgraph of the transformer architecture is used. The context features of the input sentence's words are generated by the subgraph. It also takes into account the identity and sequence of all the other words. At each word position, the element-wise sum of that representation is calculated and turned into a vector with a fixed length that represents the encoded sentence. While generating sentence embedding using DAN models, both input embedding for words and bigrams are averaged and given as input to feedforward DNN (Deep Neural Network). And the model adopted in this research is used to classify the new tweets.

## 4. Dataset and the Proposed Model

### 4.1. Sentiment Classification Model Framework

**Figure 2:**
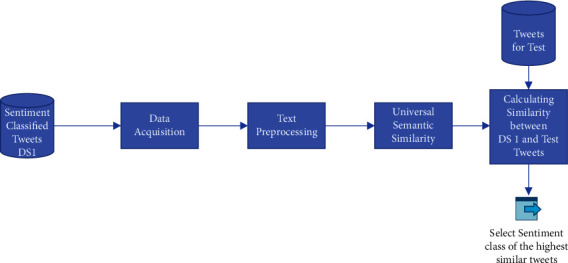
Sentiment classification framework.

The architecture of the suggested sentiment classification model for detecting the sentiment class using Universal Sentence Encoder is shown in this section. Data collection, document preprocessing, deep learning components, and semantic similarity based on Universal Sentence Encoder are all part of the platform. Each process generates an output that can be sent to the user or moved onto the next process in the sentiment classifier pipeline until it reaches the end. The semantic classification pipeline has five stages, as shown in Figure 2. Manual sentimentally categorized tweets in intermediate storage are the initial stage. The management of tweets is handled in the second stage. Format handling and extracting a relevant portion of text from a tweet are both parts of the handling procedure. It also analyses input tweets for simple and advanced text preparation. The semantic similarity model based on Universal Sentence Encoder mentioned before is established in the third stage. The final stage involves computing the similarity between the manually categorized tweets and the tested tweets and assigning the sentiment class of tweets with the highest similarity. Finally, it assigns the tested tweet to a sentiment class.

### 4.2. Dataset

We used data from Twitter in this study, as well as hand tagging that had been performed during the COVID-19 lockdown. The dataset contains 10000 tweets that have been cleansed and include themes such as COVID-19, coronavirus, and lockdown. The dataset contains names and usernames; however, they have been given codes to protect their privacy. The other columns are as follows:

1. Location
2. Tweeting time
3. Original tweet
4. Sentiment class

The sentiment class domain is (0, 1, and 2), with 0 denoting negative, 1 denoting neutral, and 2 denoting positive. The dataset is accessible for download and analysis at https://www.kaggle.com.

### 4.3. Data Analysis

**Figure 3:**
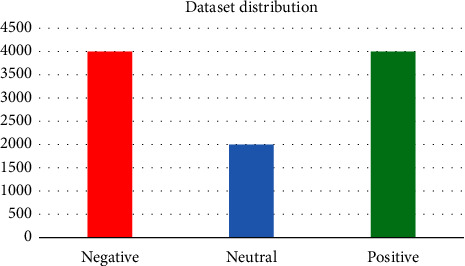
Dataset distribution.

Figure 3 shows the distribution of tweets gathered between March 1, 2020, and May 1, 2020, with the text categorized as negative, neutral, or positive. The authors manually code the sample tweets into three sentiment categories, with each sentiment mapped from 0 to 2 (negative: 0, neutral: 1, and positive: 2).  Table 1 shows some examples of tweets expressing emotions.

### 4.4. Data Preprocessing

We used natural language processing (NLP), which is a type of machine learning that aids with tweet processing. Typically, NLP employs several text mining techniques, such as the removal of noise, which entails removing stop and buzz words. Immaterial substances, such as accent marks, mathematical properties, and new-line characters, were also removed to improve the semantic similarity model performance evaluations. By removing these pieces, the size of the test universe of possible capabilities is reduced, and the degree of execution is improved.

## 5. Results Analysis

To make sure that the classification model on the dataset cannot overfit, the dataset was divided into two parts: training and testing. Trained tweets aided in the identification of patterns in data, as well as the reduction of error rates, and the testing of the dataset was used to measure model performance. 87.5 percent of tweets were used for training reasons, while 12.5 percent were used for testing purposes.  Table 2 shows the demographic information for tweet categorization.

To improve accuracy, we ran our deep learning neural network on several epochs. Hyperparameter “number of epochs” determines how many times the learning algorithm will iteratively examine the entire training dataset. Once per epoch, the internal model parameters were updated based on data from each sample in the training dataset. We ran our experiment on the epochs from 1 to 100. The accuracy of epochs 10, 20, 30, 40, 50, and 60 is shown in Table 3.

We found that after 60 epochs, the improvement in the accuracy was very little, and the best result of accuracy achieved by the model was 78.0624%.

More comparisons with different machine learning classifiers were applied to the same dataset against our semantic similarity proposed model shown in Table 4. The applied algorithms were logistic regression, fast tree, maximum entropy, fast forest, and light GBM classification. The experiment used a bag-of-words method to get the features from each tweet. The traditional term frequency-inverse document frequency (TF-IDF) method was used to get the highest words that had an impact on each sentiment class. These words were used in the training after applying the preprocessing phase. In the preprocessing phase, stop word removal, data cleaning, and stemming functions were applied.

From Tables 3 and 4, we notice that the semantic similarity model based on sentence embedding yielded accuracy results better than many traditional ML classifiers and comparable to the highest accuracy obtained.

Another experiment was used to compare the result of the proposed model with a deep learning classifier based on the CNN, and the TF-IDF was the text representation method used. The basic architecture of the CNN is listed in Table 5, while Table 6 compares the results obtained from the proposed Universal Sentence Encoder model and the CNN-based model.

**Figure 4:**
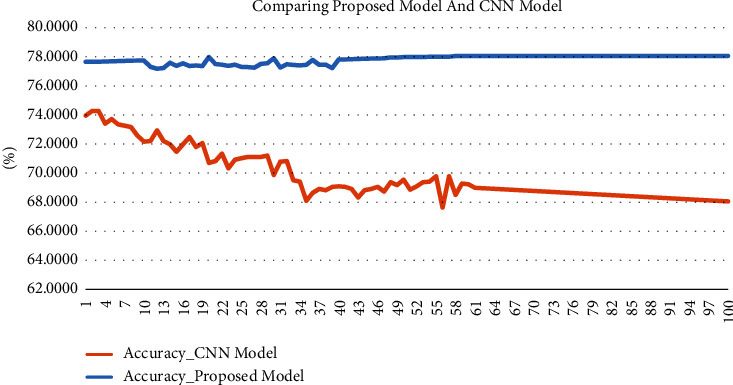
Accuracy result comparison between the proposed model against the CNN-based model for epochs from 1 to 100.

It can be noticed that the best accuracy obtained by the CNN classifier appears only with the first 10 epochs, while the accuracy decreases by increasing the number of epochs, and this is unlike a visible increase in accuracy with every increase in the number of epochs with the proposed model.  Figure 4 shows the accuracy result comparison between the proposed universal semantic similarity model against the CNN-based model for epochs from 1 to 100.

It can be noticed that the best accuracy resulting from the proposed semantic similarity classifier was 78.06% at epoch 60, while the best accuracy of the model based on CNN was 74.27% at epoch 3.

## 6. Conclusion and Future Work

In this research study, a new sentiment classification model was proposed. This model was based on the semantic similarity of tweets. Universal Sentence Encoder was used to encode the tweet's sentences into embedding vectors, and then, at the testing phase, it was used to classify new tweets based on cosine similarity. The classification accuracy reached 78.06% in the collected data, and this outperformed many ML classifiers. Also, the proposed model was compared against a CNN classifier model based on TF-IDF, and the results obtained proved that the proposed model outperformed the CNN classifier. In future work, we recommend increasing the number of tweets for training and testing, and this may enhance the obtained accuracy as the model learning rate increases with the increase in the number of training data.

## Figures and Tables

**Table 1:** Dataset sample.

Screen name	Tweets	Sentiment	Label
**89907**	@TartiiCat Well new/used Rift S are going for $700.00 on Amazon rn although the normal market price is usually $400.00. Prices are really crazy right now for VR headsets since HL Alex was announced and it's only been worse with COVID-19	0	Negative
**48751**	@MeNyrbie @Phil_Gahan @Chrisitv https://t.co/iFz9FAn2Pa and https://t.co/xX6ghGFzCC and https://t.co/I2NlzdxNo8	1	Neutral
**80443**	We've updated our amp The Essentials page What s new Latest NDIS updates changes to community shopping hours amp a list of free online activities for the whole family new videos coming soon	2	Positive

**Table 2:** Demographic representation of tweet classification.

Sentiment label	Code	Data type	
Negative	0	Training	3500
		Testing	500

Neutral	1	Training	1750
		Testing	250

Positive	2	Training	3500
		Testing	500

**Table 3:** Accuracy of the proposed model for each epoch experiment.

Epochs	Accuracy
**10**	77.742%
**20**	77.982%
**30**	77.902%
**40**	77.982%
**50**	77.9823
**60**	78.0624

**Table 4:** Accuracy of ML classifiers with TF-IDF.

Classifiers	Accuracy
Fast tree	57.59%
Fast forest	63.69%
Light GBM	50.74%
Maximum entropy	78.90%
Logistic regression	78.83

**Table 5:** Architecture of CNN.

Layer (type)	Output shape	Parameter#
Dense (dense)	(None, 1024)	61669376
Activation (activation)	(None, 1024)	0
Dropout (dropout)	(None, 1024)	0
Dense_1 (dense)	(None, 3)	3075
Activation_1(activation)	(None, 3)	0

**Table 6:** Proposed model vs. CNN classifier based on TF-IDF.

Epochs#	Proposed model		CNN model	
	Accuracy	Loss	Accuracy	Loss
**10**	77.742%	0.8005	72.16%	1.1315
**20**	77.982%	0.8006	70.70%	1.4414
**30**	77.902%	0.7993	69.87%	1.6880
**40**	77.982%	0.7994	69.09%	2.2059
**50**	77.9823	0.7987	69.55%	2.4477
**60**	78.0624	0.7991	69.23%	2.8105

## Data Availability

The data supporting the current study are available from the corresponding author upon request.
